# Editorial: Critical debates on quantitative psychology and measurement: Revived and novel perspectives on fundamental problems

**DOI:** 10.3389/fpsyg.2025.1661765

**Published:** 2025-10-16

**Authors:** Jana Uher, Jan Ketil Arnulf, Barbara Hanfstingl

**Affiliations:** ^1^School of Human Sciences, University of Greenwich, London, United Kingdom; ^2^BI Norwegian Business School, Oslo, Norway; ^3^Norwegian Defence University College, Oslo, Norway; ^4^Department of Psychology, University of Klagenfurt, Klagenfurt, Austria

**Keywords:** measurement, quantitative psychology, psychometrics, language models, ontology, epistemology, methodology, semantics

This Research Topic presents novel and revived perspectives on the fundamental problems underlying psychology's crises in replicability, validity, generalisability and thus, confidence in its findings. Our 15 articles present critical analyses of established theories and practices that are widely used in quantitative psychology and psychological ‘measurement'. They show that, contrary to current beliefs, questionable research practices (QRPs) are just surface-level symptoms of much more profound issues that are still hardly discussed.

Uher et al. argue that psychology's crises are rooted in the Questionable Research Fundamentals (QRFs) of many of its theories, concepts, approaches and methods (e.g., of psychometrics)—and therefore cannot be tackled by just remedying Questionable Research Practices (QRPs) as currently believed. The authors emphasise that advancing psychology's theories and philosophies of science is essential for integrating its fragmented empirical database and lines of research. To give new impetus to the current debates, they provide a comprehensive multi-perspectival review of key problems in psychological measurement, highlighting diverse philosophies of science (ontologies, epistemologies and methodologies) that are used in quantitative psychology and pinpointing four major areas of development.

Luchetti explores psychological ‘measurement' from a philosophical viewpoint. He notes that, without independent ways for assessing whether a given procedure does, indeed, allow for measuring the intended target property, measurement inherently involves epistemic circularity. From both a modern and a historically-situated perspective, he analyses how Fechner tackled this problem in psychophysics. He shows that Fechner developed a first successful step of a longer-term quantification process. Nevertheless, findings about individuals' sensory perceptions of physical stimuli (e.g., sounds) cannot be generalised to perceptions of all psychical phenomena in lack of evident observable properties that can be related to the psychical phenomena of interest. The author discusses epistemic circularity as a useful conceptual tool to reflect on the criteria by which measurement standards are regarded as successful in a scientific community.

Kuhbandner and Mayerhofer evaluate limitations of experimental psychology. They critically discuss the field's common assumption that the complexity of the human psyche could be studied in experimentally controlled settings, enabling the identification of law-like behaviours reflective of isolated psychical ‘mechanisms'. The authors highlight that even minimal differences in the experimental setup, including differences regarded as irrelevant for a given study, can build up to large unsystematic effects. Moreover, the identification of isolated ‘mechanisms', if such were possible, could have no explanatory value given that the psyche functions as a holistic system. They emphasise that the non-mechanistic functioning of higher-order psychical processes cannot be studied experimentally.

Similarly, Mayrhofer et al. interpret the replication crisis primarily as a symptom of an epistemological crisis derived from the mismatch of psychology's quantitative methods with the ontic nature of the psyche. They highlight that failure to replicate findings does not seem to advance the discipline by means of Popperian falsification, yet it also does not bring about Kuhnian paradigm shifts. However, it might address what Lakatos termed the ‘hard core' of the discipline's research program. Specifically, the authors argue that over-reliance on quantification in psychology entails a failure to conceptualise its methodological core. A possible solution should aim at a non-quantitative description of psychology's study phenomena that accounts for their observable but unstably quantifiable nature.

In line with this, Linkov, argues that pure (‘qualitative') mathematics might be an alternative to measurement. He contends that, in most countries, schools educate students to believe that mathematics equals quantification. Mathematics, however, is the science of abstract structure. Pure mathematics, for example, is the study of mathematical concepts. Its qualitative nature is often turned into quantification and numbers in applied technologies, which can lead to problematic concepts of measurement. Linkov argues that better public understanding of pure mathematics might help the scientific community to distinguish more clearly between qualitative pattern descriptions, quantification and numbers as well as to tackle the ensuing challenges to understanding measurement.

Scharaschkin elaborates similar views in the context of educational assessment. He critically discusses the common conceptualisation of person abilities as latent quantities, as done in many theories of psychological ‘measurement' that are aimed at locating a measurand at a point on that numerical continuum. The author suggests that van Fraassen's more expansive view of measurement as location in a logical space provides a more appropriate conceptual framework. Drawing on fuzzy logic and mathematical order theory, Scharaschkin demonstrates a ‘qualitative mathematical' theorisation for educational assessments of intersubjectively constructed phenomena (e.g., learner proficiency). This highlights the theory-dependent nature of valid representations of such phenomena, which need not be conceptualised structurally as values of quantities.

Scholz goes a step further and proposes Barad's agential realism as a suitable alternative philosophy of science for quantitative psychology. Contemporary views distinguish between the ontic existence of pre-existing objects of research (entity realism) and the researchers' epistemic approaches for exploring them. The author introduces agential realism, which rejects entity realism and views instead ontic existence and epistemic approaches as entangled and co-created by the researchers. Applied to quantitative psychology, agential realism necessitates the reconceptualisation of common assumptions about ‘true scores', context as independent influence factors, the researchers' independence of their objects of research as well as the conception of the research process itself.

Exploring philosophical perspectives on validity, Ramminger and Jacobs discuss the critical role of theory in understanding and evaluating validity in psychological ‘measurement'. The authors contrast three positions on validity: Cronbach and Meehl's construct validity, rooted in logical positivism; Borsboom's realist perspective, which highlights causal relationships, as well as Borgstede and Eggert's critique of validity as a concept. The authors contend that, despite their philosophical differences, all three perspectives converge on the essential role of theory-driven approaches in psychological ‘measurement'.

Uher provides a comprehensive critique of psychology's overreliance on statistical modelling at the expense of epistemologically grounded measurement processes. She shows that statistics is not measurement because statistics deals with structural relations in data regardless of what these data represent, whereas measurement establishes traceable empirical relations between the phenomena studied and the data representing information about them. Using basic epistemic criteria and methodological principles that underlie physical measurement (e.g., traceability, coordination, calibration), she shows that, in psychological ‘measurement' (e.g., psychometrics), many researchers mistake judgements of verbal statements for measurements of the phenomena described and overlook that statistics can neither establish nor analyse a model's relations to the phenomena explored. She elaborates epistemological and methodological fundamentals for establishing genuine analogues of measurement in psychology that consider the peculiarities of its study phenomena (e.g., higher-order complexity, non-ergodicity) as well as those of its language-based methods (e.g., inbuilt semantics).

Arnulf et al. further explore the semantic perspective on the relations between data and study phenomena. They systematically analyse how and why digital language processing can predict psychometric and statistical results fairly accurately even without access to human response data. Reviewing a range of empirical publications that demonstrate this fact, the authors argue that this is because prevalent psychometric analyses capture only the semantic representation of the variables but not the empirical correlates of these variables themselves. The authors highlight that this implies a prevalent category mistake in psychology where ‘what can be said' about a phenomenon is mistaken for the phenomenon itself. The ability of technologies, such as large language models, to predict and model response statistics a priori suggests that psychology is building a semantic rather than a nomological network of variables as commonly assumed.

In their critical analysis of the use of terms in psychology, Hanfstingl et al. emphasise the importance of identifying jingle and jangle fallacies. Jingle fallacies occur when distinct psychological study phenomena are grouped under the same term, whereas jangle fallacies arise when, vice versa, the same study phenomenon is described using different terms. The authors propose a four-step procedure to detect and address issues related to these fallacies, involving problem definition, identification, visualisation and reconceptualisation of the identified fallacies. They highlight that, ultimately, addressing jingle and jangle fallacies requires collective efforts and the incorporation of diverse theories, perspectives and methodologies.

Slaney et al. explore the rhetorical language commonly used in scientific discourse about the theory, validity and practice of psychological ‘measurement'. They examine various discourse practices, such as rhetoric (e.g., persuasion), tropes (e.g., perfunctory claims), metaphors and other ‘literary' styles as well as ambiguous, confusing or unjustifiable claims. Using conceptual analysis and exploratory grounded theory, they analysed a sample of *N* = 39 articles that were randomly selected from larger article databases representing issues published in 2021 in APA journals across a range of subject categories. The authors identify relevant themes, illustrated with constructive and useful but also misleading and potentially harmful discourse practices.

Using a more classical approach, Reisenzein and Junge introduce a framework to study the intensity of emotions that is based on a realist view of quantities and that combines modern psychometric (latent-variable) approaches with a deductive order of inquiry for testing measurement-theoretical axioms. It relies on Ordinal Difference Scaling (ODS), a non-metric probabilistic indirect scaling technique originally developed to assess sensations, bodily feelings and mental states. The authors discuss the psychological processes involved, including the comparison of stimulus intensities and the role of statistical models in ensuring measurement reliability. The approach bridges theoretical assumptions and empirical methodologies and offers insights for improving the precision of emotion-related assessments.

Brauner, in turn, takes a pragmatic and interesting step away from the necessity to measure purported ‘latent constructs'. Instead, he proposes to include several, disparate assessment points in so-called ‘micro scenarios' as an integrative contextual method to evaluate mental models and public opinion. He explains how public opinion can be mapped across people and problem spaces, offering practical examples from high-risk technologies (e.g., nuclear power). This approach offers a tool for more informed decision-making, such as in technology development and policy-making.

Paredes and Carré are also concerned with the problems of psychometrics and how these can be remedied. Whereas most approaches focus on statistical and technical best practices for researchers, the authors focus on the challenges that arise from the human-based generation of psychological data. They emphasise the necessity to develop a wider and more nuanced understanding of how different people, communities and cultures interpret and use psychometric ‘scales'. Therefore, they propose participatory approaches involving a broader group of stakeholders throughout the measurement process—including researchers, practitioners and the participants themselves.

With our compilation of research papers, we aim to contribute to and stimulate critical debates on quantitative psychology and measurement. We hope that the revived and novel perspectives discussed in these papers will provide good food for thought to motivate and help psychologist to tackle the current challenges and advance psychology as a science.

